# Preparation of Herbal Extracts for Intestinal Immune Modulation Activity Based on *In Vitro* Screening and *In Vivo* Evaluation of *Zingiber officinale* Rosc. Extracts

**DOI:** 10.3390/molecules28186743

**Published:** 2023-09-21

**Authors:** Su Ji Min, Sung Jin Kim, Jun Yeon Park, Chang-Seob Seo, You-Kyong Choi

**Affiliations:** 1 College of Korean Medicine, Gachon University, Seongnam 13120, Republic of Korea; 2 Department of Food Science and Biotechnology, Kyonggi University, Suwon 16227, Republic of Korea; 3 KM Science Research Division, Korea Institute of Oriental Medicine, Daejeon 34054, Republic of Korea

**Keywords:** IgA, intestinal immune modulation, Peyer’s patch, *Zingiber officinale* Rosc.

## Abstract

Ten traditional herbal extracts effective against diarrhea, infectious diseases, and bacterial activity were selected and analyzed for Peyer’s patch cell-mediated intestinal immunomodulatory activity *in vitro* and *in vivo*. Among the 10 herbal extracts, *Zingiber officinale* Rosc. (ZO) extract induced the highest secretion of immunoglobulin A (IgA) and granulocyte macrophage colony-stimulating factor (GM-CSF) in the cells of Peyer’s patches. Furthermore, animal experiments showed that IA production was enhanced with the oral administration of ZO extract (100 mg/kg and 300 mg/kg) for 10 days. In addition, 6-, 8-, 10-gingerol, and 6-, 8-, 10-shogaol, the six major index compounds of ZO extract, were analyzed using HPLC. Our study findings confirm the intestinal immunomodulatory activity of ZO extract and lay a strong foundation for future analytical studies aimed at determining the active components of ZO extracts.

## 1. Introduction

The epithelial layer of the intestinal mucosa is continuously exposed to foreign substances or hormones passing through the intestinal tract. The adult intestinal mucosa, which has an area 200 times larger than that of the skin surface, is the site of contact with various antigens [1,2]. Mucosa-associated lymphatic tissues (MALTs) are divided into various mucosal lymphatic organs of the body, such as nasal-associated lymphatic tissue (NALT) and bronchus-associated lymphatic tissue (BALT), and they play an important role in immune defenses [3,4]. Among these mucosal lymphoid organs, gut-associated lymphoid tissue (GALT) represents the largest lymphoid tissue *in vivo* and is composed of Peyer’s patches; moreover, it plays a pivotal role in the digestion and absorption of numerous essential nutrients, simultaneously serves as a barrier against pathogenic microorganisms and harmful substances, and is known as an induction site [5,6]. The intestinal mucosa contains more than 80% of activated B cells, which eventually differentiate into plasmablasts and plasma cells, making the intestine the largest antibody-producing structure in the body [7]. In addition, Peyer’s patches contain specialized M cells that are responsible for transporting intestinal antigens, and the basolateral membrane of M cells contains B lymphocytes, T lymphocytes, macrophages, and dendritic cells [7]. Reports have indicated that the activation of Peyer’s patch cells by intestinal antigens leads to the production of cytokines that activate B lymphocytes to differentiate into plasma cells and induce immunoglobulin A (IgA) secretion [8,9]. Secreted IgA promotes the involvement of eosinophils and macrophages in systemic immune responses [10]. Thus, Peyer’s patches play a major role in the activation of the systemic immune system.

Herbal medicines, including *Amomum kravanh* Pierre ex Gagnep., *Schisandra chinensis* Baillon, *Panax ginseng* C. A. Meyer, *Zingiber officinale* Rosc. (ZO), *Glycyrrhiza uralensis* Fisch., *Scutellaria baicalensis* Georgi, *Paenoia suffruticosa* Andrews, and *Coptis chinesis* Franch. have been widely prescribed to treat recurrent diarrhea or infectious diseases [11,12,13,14]. In addition, medications such as *Paeonia suffruticosa* Andrews, *Spatholobus suberectus* Dunn, and *Salvia miltiorrhiza* Bunge have been used to eliminate blood stasis [11,12,13]. Many studies have focused on the anti-inflammatory activities of these natural products, such as *Coptis chinesis* Franch., *Glycyrrhiza uralensis* Fisch., *Salvia miltiorrhiza* Bunge, *Schisandra chinensis* Baillon, *Paeonia suffruticosa* Andrews, and *Spatholobus suberectus* Dunn [15]. Recently, several reports have indicated that natural product-derived extracts such as red pepper capsaicin extract [16], garlic extract [17], red ginseng-derived polysaccharides [18], citrus peel-derived pectin polysaccharides [19], mushroom polysaccharides [20], and cell-wall extracts from lactic acid bacteria [21,22] activate Peyer’s patch cell-mediated intestinal immunity and the systemic immune system. Therefore, in this study, we screened Peyer’s patch-mediated IgA and granulocyte macrophage colony-stimulating factor (GM-CSF) production using individual extracts from ten natural products used as herbal medicines. Among them, ZO extract exhibited the highest IgA and GM-CSF production. Therefore, we investigated its intestinal immune modulation activity using animal experiments and analyzed the six major index compounds 6-, 8-, and 10-gingerol, and 6-, 8-, and 10-shogaol.

## 2. Results and Discussion

### 2.1. HPLC Profiling of Six Index Compounds of ZO Extract

**Figure 1:**
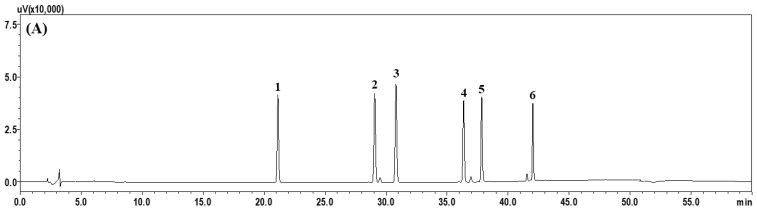

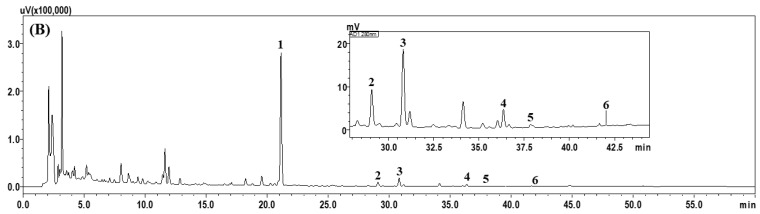
HPLC chromatogram of the standard mixtures (**A**) and ZO extract (**B**) monitored at a UV wavelength of 280 nm. 1: **6**-Gingerol. 2: **8**-Gingerol. 3: **6**-Shogaol. 4: **10**-Gingerol. 5: **8**-Shogaol. 6: **10**-Shogaol.

In this study, we performed the simultaneous HPLC-based profiling of six phenolic compounds (6-, 8-, and 10-gingerol, and 6-, 8-, and 10-shogaol) representing the major components of ZO. These compounds were well separated within 45 min without overlapping with other neighboring peaks, and they were detected at 21.13, 29.07, 30.80, 36.35, 37.83, and 42.03 min (Figure 1). Quantification was performed at 280 nm, which corresponds to the maximum UV absorption wavelength. For quantitative analysis, calibration curves were drawn at 1.56−100.00 μg/mL (6-gingerol) and 0.31−20.00 μg/mL (8-gingerol, 6-shogaol, 10-gingerol, 8 shogaol, and 10-shogaol), respectively. The coefficients of determination of these components were ≥0.9997, thus showing good linearity. The six major components of ZO were simultaneously quantified with optimized HPLC analysis conditions, and the results are presented in Table 1. Among them, 6-gingerol was the most abundant, at 6.64 mg/g.

### 2.2. Effects of the Ten Herbal Medicine Extracts on Peyer’s Patch Cell Proliferation in Ex Vivo Experiments

**Figure 2:**
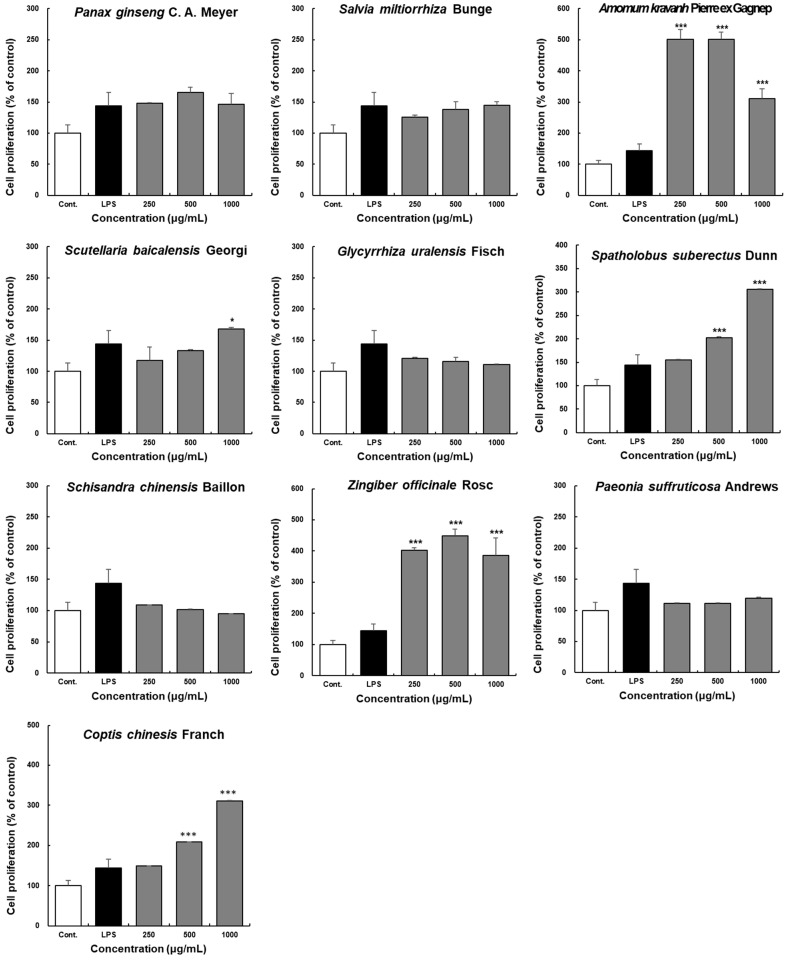
Effects of the ten herbal extracts on the proliferation of Peyer’s patch cells in C3H/HeN mice. Peyer’s patch cells were seeded into 96-well plates at 4 × 10^5^ cells/well density. The ten extracts were administered at the indicated concentrations (250, 500, and 1000 μg/mL) for 4 days. Cell viability was determined using the EZ-Cytox cell viability assay kit. Data are presented as the means ± SDs of triplicate experiments. *** *p* < 0.0001 compared with the control groups. * *p* < 0.01 compared with the control groups.

Peyer’s patches are important lymphoid organs in the intestinal tract that contain B cells, T cells, macrophages, and dendritic cells [23,24]. The proliferative ability of Peyer’s patches increases the number of these immune cells and helps activate the intestinal immune system [3,4,5]. The present study aimed to characterize the effects of ten extracts on intestinal immune system activity mediated by Peyer’s patch cell proliferation in C3H/HeN mice. As shown in Figure 2, among the groups treated with the extracts isolated from the ten herbal medicines, Peyer’s patch cell proliferation was significantly increased in the groups treated with ZO and *Amomum kravanh* Pierre ex Gagnep. extracts at 250 and 500 μg/mL compared with the control group. However, 1000 μg/mL ZO and *Amomum kravanh* Pierre ex Gagnep. extract treatment reduced proliferation, and there was no cytotoxicity. *Coptis chinesis* Franch. also promoted cell proliferation at 500 and 1000 μg/mL concentrations, and *Spatholobus suberectus* Dunn significantly increased proliferation at 1000 μg/mL. However, cell proliferation was not affected by *Panax ginseng* C. A. Meyer, *Salvia miltiorrhiza* Bunge, *Scutellaria baicalensis* Georgi, *Glycyrrhiza uralensis* Fisch., *Schisandra chinensis* Baillon, and *Paeonia suffruticosa* Andrews extracts. None of the studied extracts induced cellular toxicity in Peyer’s patches.

### 2.3. Effects of Ten Herbal Medicine Extracts on IgA Production by Peyer’s Patch Cells Ex Vivo 

IgA induced in Peyer’s patches inhibits the adhesion of various harmful foreign substances and pathogenic microorganisms to the intestinal tract and prevents antigens from being absorbed by the mucosal surface [3,5]. In addition, it is known to have antibody activity against various bacteria or viruses and thus plays an important role in the immune response of the mucosal surface [6]. Peyer’s patch cell-mediated IgA production is driven by cytokines, such as IL-6 and IL-5, which play important roles in the functional control and signal transduction of other immune cells [5]. A recent report indicated that IgA secretion by Peyer’s patch cells was increased by extracts of various herbal preparations and that IL-6 was produced by dendritic cells in Peyer’s patches, thereby inducing IgA secretion [7]. Therefore, IgA production in the intestine can be considered an indicator of immune response. 

**Figure 3:**
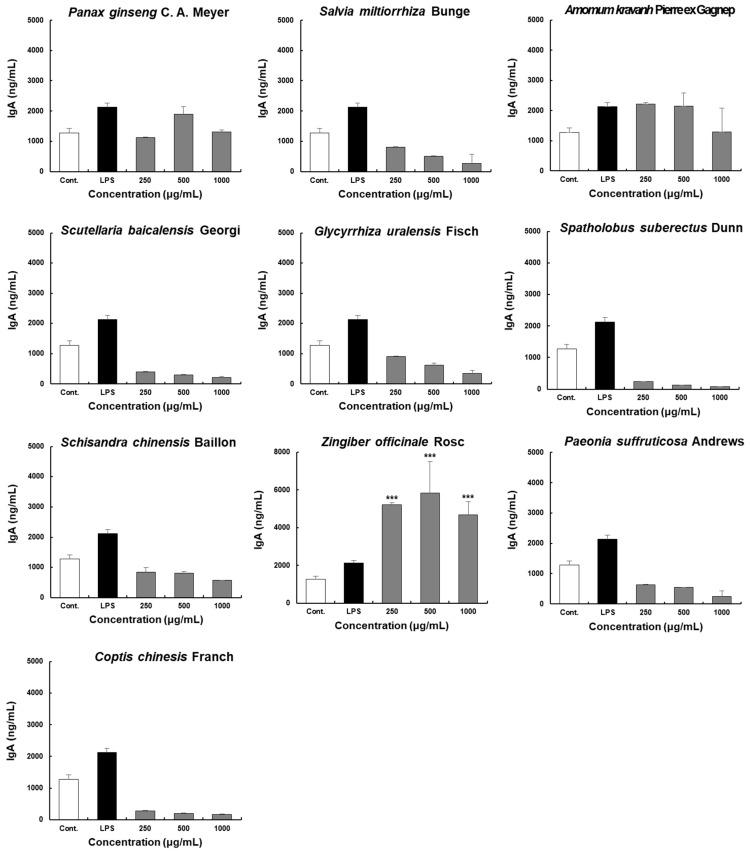
Effects of the ten herbal extracts on IgA secretion by Peyer’s patch cells in C3H/HeN mice. Peyer’s patch cells were seeded into 96-well plates at 4 × 10^5^ cells/well density. The ten extracts were incubated at the indicated concentrations (250, 500, and 1000 μg/mL) for 4 days. Subsequently, the cell supernatant was harvested for IgA analysis using commercial ELISA kits. Data are presented as the means ± SDs of triplicate experiments. *** *p* < 0.0001 compared with the control groups.

Treatment with LPS, a positive control for IgA production in Peyer’s patch cells, significantly increased IgA production, as shown in Figure 3. Among the ten extracts, ZO extract increased IgA production in a concentration-dependent manner and led to approximately 1.8-fold higher IgA secretion than that obtained with the positive control at 125 μg/mL. The highest IgA production was observed following treatment with 500 μg/mL ZO. IgA production in the groups treated with the ZO and *Amomum kravanh* Pierre ex Gagnep. extracts were consistent with that of the positive control group. However, IgA production induced by *Salvia miltiorrhiza* Bunge, *Scutellaria baicalensis* Georgi., *Glycyrrhiza uralensis* Fisch., *Spatholobus suberectus* Dunn, *Schisandra chinensis* Baillon, *Paeonia suffruticosa* Andrews, and *Coptis chinesis* Franch. extracts were lower than that of the control group. Collectively, ZO induced maximum stimulation of intestinal immune activity by promoting IgA production *in vitro*.

### 2.4. Effects of the Ten Extracts on GM-CSF Production by Peyer’s Patch Cells in Ex Vivo Experiment

Cytokines are soluble proteins secreted by immune cells and play an important role in the immune system. In addition, they modulate signal transduction in immune cells and impact the entire immune system by forming a complex cytokine network [8,9]. Herbal extracts can activate the T cells of Peyer’s patches to produce cytokines, such as GM-CSF, which are necessary for the maturation and differentiation of hematopoietic cells and the proliferation of bone marrow cells [25,26]. Peyer’s patches are a known source of CSF and various cytokines [7,27]. Thus, activated T cells can affect the secretion of the hematopoietic growth factor GM-CSF in Peyer’s patches [28]. 

**Figure 4:**
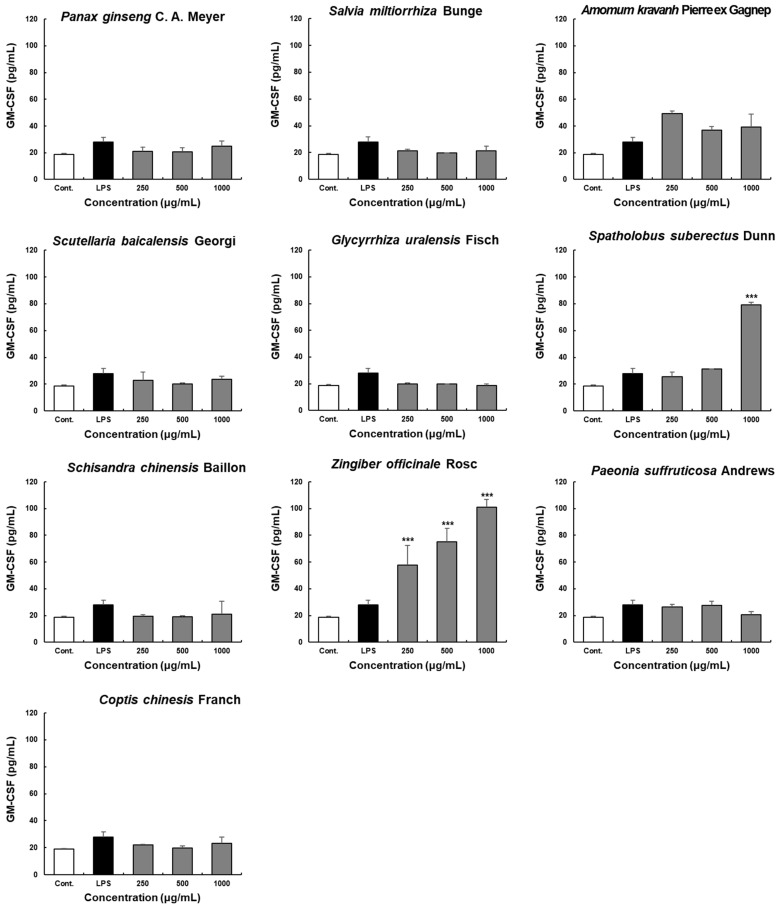
Effects of the ten herbal extracts on GM-CSF secretion by Peyer’s patch cells in C3H/HeN mice. Peyer’s patch cells were seeded into 96-well plates at 4 × 10^5^ cells/well density. Ten extracts were administered at various concentrations (250, 500, and 1000 μg/mL) for 4 days. Subsequently, the cell supernatant was harvested for GM-CSF analysis. GM-CSF secretion was determined using commercial ELISA kits. Data are presented as the means ± SDs. *** *p* < 0.0001 compared with the control groups.

GM-CSF production by Peyer’s patches was determined in the culture supernatants of the ten extracts (Figure 4). Among the ten herbal extracts, only ZO extract increased GM-CSF production at all tested concentrations, whereas treatment with *Amomum kravanh* Pierre ex Gagnep. extract increased GM-CSF production only slightly. Therefore, the results show that ZO extract affected GM-CSF production as well as IgA production. 

Collectively, these results indicate that ZO extract promotes the secretion of GM-CSF in Peyer’s patches in the intestinal immune system, and these cytokines act as hematopoietic growth factors. Cytokine secretion plays an important role in modulating the activity of circulatory immune cells. GM-CSF secretion via activation of Peyer’s patch cells can not only induce bone marrow cell proliferation but also contribute to the activation of the systemic immune system.

### 2.5. Determination of Peyer’s Patch Cell Proliferation and IgA Production Induced by Six Major Index Compounds Present in ZO Extract 

The six major index compounds of ZO (6-, 8-, and 10-gingerol, and 6-, 8-, and 10-shogaol) were investigated to reveal their effect on the production of IgA and GM-CSF. We first analyzed the viability of Peyer’s patch cells using an EZ-Cytox assay. As shown in Table 2 (upper table), cells treated with LPS showed significantly increased proliferation compared with that of the control group. However, the viability of cells treated with 6-, 8-, and 10-gingerol, and 6-, 8-, and 10-shogaol was not affected. 

Next, we analyzed IgA production in Peyer’s patch cells after treatment with the six major compounds. As shown in Table 2 (lower table), LPS significantly increased IgA production compared with the control. In contrast, treatment with 6-, 8-, and 10-gingerol, and 6-, 8-, and 10-shogaol did not induce IgA production. As shown in Figure 2 and Figure 3, IgA and GM-CSF production mediated by ZO extract was not regulated by the major index compounds and thus was likely controlled by other substances. 

### 2.6. Effects of ZO Extract Administered Orally on Mouse IgA Production

**Figure 5:**
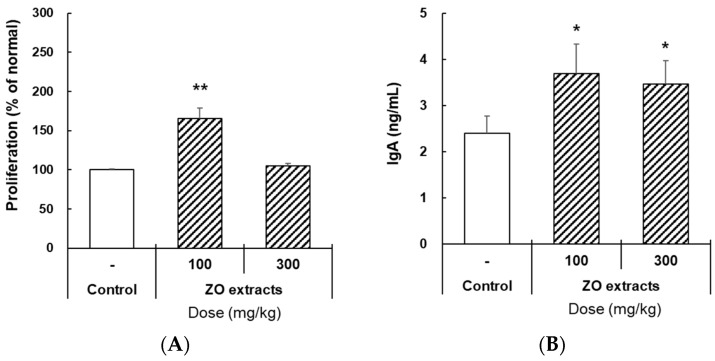
Effects of oral administration of ZO extract on mouse IgA production. (**A**) Five C3H/HeN mice per group were orally and daily administered with the indicated doses of ZO extract for 10 days. (**A**) Mice were sacrificed 11 days after oral treatment, and Peyer’s patches of the small intestine were then collected. Then, immune cells isolated from Peyer’s patches were incubated for 4 days. Cell viability was determined using the EZ-Cytox cell viability assay kit. (**B**) Mice were sacrificed 11 days after oral treatment, and Peyer’s patches of the small intestine were then collected. Then, immune cells isolated from Peyer’s patches were incubated for 4 days. Subsequently, the cell supernatant was harvested for IgA analysis. IgA secretion was determined using commercial ELISA kits. Data are presented as the means ± SDs of three independent experiments. * *p* < 0.01 vs. the control group. ** *p* < 0.001 vs. the control group.

Based on the *in vitro* results, we next investigated the *in vivo* intestinal immunomodulation activity of ZO extract. In our previous study [18], activation of intestinal immune modulating activity in mice was confirmed after oral administration of natural polysaccharides (5–50 mg/kg). Therefore, in this study, crude extract of ZO was orally and daily administered to C3H/HeN mice for 10 days at 100 and 300 mg/kg. During the oral administration of ZO extract, we measured the bodyweight of each mouse every 2–3 days. The bodyweight of mice in all the groups increased slightly. To verify the intestinal immune modulating activity of ZO extract, we measured lymphocyte proliferation and IgA production by Peyer’s patches after oral administration. As shown in Figure 5A, the proliferation of immune cells in Peyer’s patches slightly increased in the 100 mg/kg ZO group but did not significantly change in the 300 mg/kg group. On the other hand, the secretion of IgA by Peyer’s patches was significantly increased in the ZO-treated group compared with the control group (Figure 5B). Hereby, this suggests that the switching of B cells to plasma cells is more important than proliferation for IgA secretion. Collectively, these results may indicate that macromolecules present in ZO extract, such as polysaccharides, rather than the six major index compounds, augmented IgA production in Peyer’s patches and may affect intestinal immune modulating activity in mice.

## 3. Materials and Methods

### 3.1. Preparation of Crude Extract from Herbal Medicines

Extracts of ten herbal medicines (15 g each) were prepared using 150 mL of distilled water at 100 °C for 60 min. The extracts were then filtered through nonwoven fabric, and 120 mL of herbal extract was ultimately obtained for each medicine. The extracts were freeze-dried, and the yield was calculated by measuring the dried amount (Table 3). These extracts were then used for *in vitro* assays, and ZO extract, which showed best immunomodulatory activity *in vitro*, was orally administered to mice to evaluate its intestinal immune modulating activity *in vivo*.

### 3.2. Chemicals and Reagents

**Figure 6:**
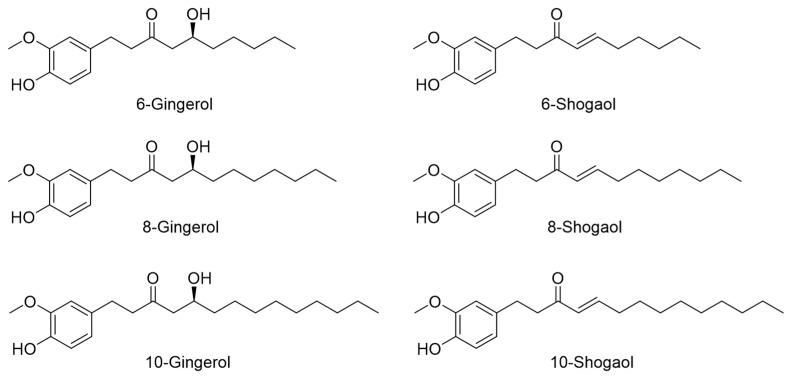
Chemical structures of six major index compounds in ZO.

The following six reference standards (Figure 6) were used for the phytochemical analysis of ZO based on high-performance liquid chromatography (HPLC): 6-gingerol (99.3%), 8-gingerol (98.0%), 10-gingerol (98.1%), 6-shogaol (99.2%), 8-shogaol (98.9%) (all obtained from Shanghai Sunny Biotech Co., Ltd., Shanghai, China), and 10-shogaol (98.6%) (obtained from ChemNorm Biotech Co., Ltd., Wuhan, China). Solvents (methanol, acetonitrile, and distilled water of HPLC grade) for HPLC analysis were purchased from J. T. Baker (Phillipsburg, NJ, USA). Formic acid (ACS reagent grade, ≥98.0%) for mobile phase preparation was obtained from Merck (Darmstadt, Germany).

### 3.3. HPLC Profiling of the Six Major Index Compounds in ZO Extract 

The HPLC profiling of the six major phenolic compounds in ZO was conducted using a Shimadzu Prominence LC-20A system (Kyoto, Japan) and LabSolution software (Version 5.53; SP3, Kyoto, Japan). The six analytes were separated using a SunFire^TM^ C_18_ analytical column (4.6 × 250 mm, 5 μm; Waters, Milford, MA, USA) maintained at 40 °C. The mobile phase was composed of 0.1% (*v*/*v*) formic acid in distilled water (A) and 0.1% (*v*/*v*) formic acid in acetonitrile (B) under the following gradient elution conditions: 0–30 min, 30–70% B; 30–40 min, 70–100% B; 40–45 min, 100% B; 45–50 min, 100–30% B. The re-equilibration time was 10 min. The flow rate and injection volume were set to 1.0 mL/min and 10 μL, respectively. The standard stock solution of the six analytes was prepared at a concentration of 1.0 mg/mL with methanol and then diluted when used. The sample solution for quantitative analysis was ultrasonically extracted for 60 min by adding 10.0 mL of 70% methanol to 500.0 mg of lyophilized ZO. Finally, the prepared solutions were filtered through a 0.2 μm syringe filter (Pall Life Sciences, Ann Arbor, MI, USA) before injection into the HPLC instrument.

### 3.4. Animals

C3H/HeN mice (6-week-old, female) were purchased from Orientbio (Seongnam, Republic of Korea) and housed at 23 ± 2 °C with 55% ± 10% humidity under a 12/12 h light/dark cycle, and they were provided free access to a standard laboratory diet and water. For the *in vivo* study, the mice were treated for 10 days with ZO extract at 100 mg/kg and 300 mg/kg using oral gavage feeding needles. Mice in the control group were administered sterilized distilled water. All animal experiments were performed in accordance with the Institutional Animal Care and Use Committee (IACUC) at Gachon University (GU1-2022-IA0050).

### 3.5. Ex Vivo Proliferation of Peyer’s Patch Lymphocytes

Peyer’s patches were collected from the small intestine wall of the C3H/HeN mice. Next, the immune cells were separated using a sterilized metal mesh (100 mesh), and the cell suspension was filtered using a cell strainer (Nylon, Falcon, NJ, USA). Then, 10% fetal bovine serum, 1% penicillin–streptomycin (Gibco, Grand Island, NY, USA), and 0.1% 2-mercaptoethanol (Gibco-Invitrogen, Carlsbad, CA, USA) were mixed with RPMI 1640 (Corning, CA, USA) and used to isolate lymphocytes. The lymphocytes isolated from Peyer’s patches were seeded into a 96-well plate at a density of 4.0 × 10^5^ cells/well, treated with the extracts at various concentrations, and incubated for 4 days in an incubator set at 37 °C and supplied with 5% CO_2_. After incubation, EZ-Cytox (Dogenbio, Seoul, Republic of Korea) was added to each well, and optical density at 450 nm was determined using a microplate reader (Emax, Molecular Devices, San Jose, CA, USA). 

### 3.6. Determination of IgA and GM-CSF Production in Peyer’s Patch Cells Ex Vivo

Peyer’s patches were collected from the wall of the small intestine of C3H/HeN mice, and the immune cells were separated using a sterilized metal mesh (100 mesh). The cell suspension was then filtered using a cell strainer (Nylon, Falcon, NJ, USA) and mixed with RPMI 1640 containing FBS, penicillin–streptomycin, and 2-mercaptoethanol. The lymphocytes were seeded into a 96-well plate at a density of 4.0 × 10^5^ cells/well, treated with the extracts at various concentrations, and incubated for 4 days in an incubator set at 37 °C and supplied with 5% CO_2_. After incubation, the cell supernatant was harvested for IgA (Invitrogen, CA, USA) and GM-CSF (BD Biosciences Co., Ltd., San Diego, CA, USA) analysis using commercial ELISA kits, respectively. Briefly, IgA or GM-CSF capture antibody was diluted, and 100 µL of diluted antibody was added to each well of a 96-well plate; the plates were incubated overnight at 4 °C. The next day, excess antibody was discarded, and the plate was washed three times with washing buffer. Then, 200 µL of blocking buffer (2.5% skim milk in PBS) was added to each well, and the plate was incubated at room temperature for 1 h. The plate was then washed three times with washing buffer (PBS containing 0.1% Tween 20). Then, 100 µL of culture supernatant from Peyer’s patches was added to each well and incubated at room temperature for 2 h. Next, the plate was washed three times with washing buffer, after which diluted HRP-conjugated secondary antibody was added to each well, and the plate was incubated at room temperature for 1 h. The plate was washed three times with washing buffer, and then TMB (3,3′,5,5′–tetramethylbenzidine) solution, a substrate capable of reacting with HRP, was added to each well and reacted in the dark for 10 min. The reaction was stopped by adding 0.5 N sulfuric acid. Color development was measured at 450 nm using a microplate reader (Emax, Molecular Devices, USA).

### 3.7. Determination of IgA Production in Peyer’s Patches In Vivo

After oral administration of ZO extract for 10 days, mice were sacrificed, and Peyer’s patch cells were collected from the small intestine as described in Section 3.5 and Section 3.6. The cells isolated from Peyer’s patches were seeded on a 96-well plate at a density of 4.0 × 10^5^ cells/well and incubated for 4 days in an incubator at 37 °C and 5% CO_2_. After incubation, cell proliferation and IgA production were analyzed using EZ-Cytox and IgA ELISA kits as described in Section 3.5 and Section 3.6.

### 3.8. Statistical Analysis

The results are expressed as the means ± standard deviations of triplicate experiments. The results were analyzed statistically using the Mann–Whitney U test in GraphPad Prism 8 (GraphPad Software, San Diego, CA, USA), with *** *p* < 0.0001, ** *p* < 0.001, and * *p* < 0.01 being considered statistically significant.

## 4. Conclusions

The intestinal mucosa is the largest lymphoid tissue among the mucosal immune organs, and Peyer’s patches in the intestinal tract not only contribute to the activation of the systemic immune system by secreting various cytokines but also play a central role in local IgA production. The appropriate induction and regulation of secretory IgA can be used to treat diseases, such as intestinal infections.

We aimed to investigate the intestinal immune modulating activity of ten natural products mainly used as herbal medicine prescriptions for the treatment of inflammation and diarrhea. First, the medicines were extracted with hot water, and the extracts were freeze-dried to evaluate the intestinal immune system modulatory activity mediated by Peyer’s patches. Among the ten extracts evaluated in this study, ZO showed the highest IgA and GM-CSF production activity mediated by Peyer’s patch cells *in vitro*. Furthermore, oral administration of ZO extract enhanced IgA production in Peyer’s patches. Since Peyer’s patches are the main source of IgA production, which maintains intestinal homeostasis against intestinal infection, the induction of IgA production by ZO extract may promote intestinal health and improve diarrhea symptoms. 

Notably, the six major index compounds of ZO (6-, 8-, and 10-gingerol, and 6-, 8-, and 10-shogaol) could not activate intestinal immune modulation responses *in vitro*. These results suggest that intestinal immune activity induced by ZO extract is attributable to the crude polysaccharide fraction, which includes larger high-molecular-weight compounds. Therefore, in future studies, the active components of ZO extract that affect intestinal immune activity must be isolated and purified.

## Figures and Tables

**Table 1:** Six major compounds (mg/g) in the ZO extract using HPLC.

Compound	Amount (mg/g)		
	Mean	SD	RSD (%)
**6**-Gingerol	6.64	0.02	0.36
**8**-Gingerol	0.22	0.00	1.62
**6**-Shogaol	0.28	0.00	0.42
**10**-Gingerol	0.16	0.00	0.52
**8**-Shogaol	0.01	0.00	1.84
**10**-Shogaol	<LOQ *	-	-

* LOQ: limit of quantitation.

**Table 2:** Effects of 6-, 8-, and 10-gingerol, and 6-, 8-, and 10-shogaol on the proliferation of Peyer’s patch cells and production of IgA in C3H/HeN mice.

Viability (%)	Control		10 μM		25 μM		50 μM	
	Average	SD	Average	SD	Average	SD	Average	SD
Control	100.0	1.2	-	-	-	-	-	-
LPS	220.5 ***	8.4	-	-	-	-	-	-
**6**-Gingerol	-	-	95.3	1.7	93.8	0.4	92.1	0.7
**8**-Gingerol	-	-	96.6	1.2	93.6	1.0	90.9	0.7
**10**-Gingerol	-	-	96.8	0.2	93.9	1.3	93.2	2.4
**6**-Shogaol	-	-	94.2	1.2	88.4	5.2	94.9	2.1
**8**-Shogaol	-	-	94.0	2.4	92.7	2.6	91.4	2.0
**10**-Shogaol	-	-	96.9	0.4	94.4	1.8	95.7	2.4
**IgA (pg/mL)**	**Control**		**10 μM**		**25 μM**		**50 μM**	
	**IgA**	**SD**	**IgA**	**SD**	**IgA**	**SD**	**IgA**	**SD**
Control	3246.3	19.9	-	-	-	-	-	-
LPS	5260.4 ***	57.8	-	-	-	-	-	-
**6**-Gingerol	-	-	2951.9	142.5	2868.0	227.4	2995.1	54.3
**8**-Gingerol	-	-	2052.1	17.0	2481.6	156.1	2654.4	74.7
**10**-Gingerol	-	-	1579.4	156.1	2606.4	203.6	2767.2	234.2
**6**-Shogaol	-	-	1452.2	193.4	1672.9	84.8	1812.1	125.6
**8**-Shogaol	-	-	1622.5	54.3	2548.8	95.0	2724.0	254.5
**10**-Shogaol	-	-	1900.9	54.3	2064.1	142.5	2896.8	57.7

*** *p* < 0.001 compared with the control groups.

**Table 3:** The 10 herbal medicine extracts used in this study.

Number	Herbal Medicine	Origin	Freeze-Dried Extract (g)	Yield (%)
1	*Panax ginseng* C. A. Meyer	Republic of Korea	8.3	27.66
2	*Salvia miltiorrhiza* Bunge	China	12.2	40.6
3	*Amomum kravanh* Pierre ex Gagnep.	Indonesia	9.9	33.0
4	*Scutellaria baicalensis* Georgi	China	1.5	5.0
5	*Glycyrrhiza uralensis* Fisch.	China	11.8	39.3
6	*Spatholobus suberectus* Dunn	Vietnam	9.1	30.3
7	*Schisandra chinensis* Baillon	China	11.7	39.0
8	*Zingiber officinale* Rosc.	Peru	10.3	34.3
9	*Paeonia suffruticosa* Andrews	China	6.6	22.0
10	*Coptis chinesis* Franch.	China	10.2	34.0

## Data Availability

Not applicable.
